# Reply to “The electroencephalogram in syncope”

**DOI:** 10.1002/jgf2.217

**Published:** 2018-10-24

**Authors:** Manabu Izumi, Taro Okabe, Masayoshi Komura, Yasushi Hayashi

**Affiliations:** ^1^ Department of General Medicine Saiseikai Utsunomiya Hospital Tochigi Japan

Thank you for your interest in our paper.

As you mentioned, we did not reference Gastaut's research in this report. We researched various reports in PubMed, many of which discussed EEG and whole‐brain ischemia. However, a large portion of them discussed the relationship between relative brain ischemia, which was brought on by the head‐up tilt test and the Valsalva maneuver, and severe hypotension. As far as we could ascertain, few reports discuss EEG, convulsion, whole‐brain ischemia, and cardiac arrest. We apologize for lacking information on Gastaut's works. By searching “Author; Gastaut H” in PubMed, we found 418 reports. Eighteen reports examined the occurrence of syncope among patients with hypotension when the vasovagal reflex occurs or they perform the Valsalva maneuver.^1^ Additionally, Lempert et al^2^ reported on convulsive syncope with hypotension through videometric analysis.

Apart from us, Diaz‐Castro et al^3^ also reported a case in which cardiac arrest caused systemic epilepsy. It is still a curious phenomenon for many physicians. However, there are ethical issues in conducting experimental trials to reveal the mechanism of this phenomenon in humans. Therefore, it is very important to add new knowledge through case reports. I have confidence that this case report has enough value to be published, even if my description of the background of this case did not refer to Gastaut's works, because the cause of brain ischemia in our case is quite different from Gastaut's.

## CONFLICT OF INTEREST

The authors have stated explicitly that there are no conflicts of interest in connection with this article.
